# TLR4 Ligands in Typhoid Vi Polysaccharide Subunit Vaccines Contribute to Immunogenicity

**DOI:** 10.4049/immunohorizons.2300085

**Published:** 2024-01-05

**Authors:** Kishore R. Alugupalli

**Affiliations:** Department of Microbiology and Immunology, Sidney Kimmel Medical College, Thomas Jefferson University, Philadelphia, PA

## Abstract

Activation of B cells and T cells requires the engagement of costimulatory signaling pathways in addition to Ag receptor signaling for efficient immune responses. None of the typhoid Vi polysaccharide (ViPS) subunit vaccines contains adjuvants that could activate costimulatory signaling pathways, yet these vaccines are very immunogenic. I hypothesized that residual TLR ligands present in the ViPS preparation used for making typhoid subunit vaccines account for the robust immune response generated by these vaccines. I show the presence of endotoxin, a potent agonist of TLR4, in ViPS preparations and ViPS vaccines. Furthermore, I found that ViPS obtained from various sources induces the production of proinflammatory cytokines such as IL-6 from mouse peritoneal exudate cells. Unconjugated and tetanus toxoid–conjugated ViPS vaccines activate human and mouse TLR4. Mice deficient in TLR4 or the signaling adaptors MyD88 and Trif (Toll/IL-1R domain–containing adapter inducing IFN-β) are severely impaired in generating anti-ViPS responses to these vaccines. Elimination of the TLR4 agonist in ViPS preparation resulted in the loss of immunogenicity, and addition of lipid A, a known TLR4 agonist, restored the immunogenicity. These data highlight the importance of associated TLR ligands in the immunogenicity of ViPS subunit vaccines.

## Introduction

*Salmonella enterica* serovar Typhi (*S.* Typhi) is the causative agent of typhoid fever in humans. Global estimates reported by the Centers for Disease Control and Prevention indicate that 21.6 million cases of typhoid fever occur each year, resulting in 226,000 deaths (1). The rapid emergence of multiple drug-resistant strains of *S.* Typhi now complicates the treatment of typhoid (2). Typhoid is a vaccine-preventable disease, and vaccination of high-risk populations such as infants and young children is considered the most promising strategy for control (3). Vi polysaccharide (ViPS) of *S.* Typhi is a target for immune responses, and anti-ViPS Abs correlate with protection against typhoid fever (3–5). Two types of ViPS subunit vaccines are currently available (5): plain ViPS or unconjugated ViPS; for example, Typhim Vi, a U.S. Food and Drug Administration–approved vaccine, induces a T cell–independent B cell response, and its efficacy is ∼55% in older children and adults, with the immunity conferred being short-lived (5–7). Importantly, plain ViPS vaccines do not induce Ab responses in children <2 y of age. The other type of subunit vaccines are referred to as polysaccharide and protein conjugate vaccines, which induce T cell–dependent B cell responses across all ages. The typhoid conjugate vaccines (TCVs) are comprised of ViPS chemically coupled to a variety of carrier proteins, for example, recombinant exoprotein A from *Pseudomonas aeruginosa* (8), CRM197, a nontoxic mutant of diphtheria toxin (9), tetanus toxoid (10), or diphtheria toxoid (11). Typbar TCV (ViPS conjugated to tetanus toxoid), the first World Health Organization prequalified vaccine, is very safe and induces a long-lasting Ab response in infants and young children, with ∼80% efficacy in disease endemic areas, for example, Malawi, Bangladesh, and Nepal (12–14).

Adaptive immune responses require costimulatory signals in addition to the signals generated by B and T cell Ag receptors (15). Stimulation of TLRs triggers the induction of costimulatory molecules, amplifies B cell activation, promotes dendritic cell maturation, and increases Ag presentation to T cells (16). TLR ligands help direct adaptive immune responses to Ags derived from microbial pathogens, and many vaccines incorporate TLR ligands as adjuvants to augment Ag-specific responses (17). However, none of the ViPS vaccines incorporates adjuvants, yet they are very immunogenic. ViPS used in making typhoid subunit vaccines is isolated from directly from Gram-negative bacteria, for example, *S. *Typhi, therefore it may contain trace amounts of bacterial LPS (also known as endotoxin), a potent TLR4 ligand. In fact, many commercial vaccines contain various amounts of LPS/endotoxin, and the levels of LPS/endotoxin in the same vaccine vary from lot to lot (18–20). In the current study I tested the hypothesis that associated LPS in ViPS preparations acts as an adjuvant and contributes to the immunogenicity of the typhoid subunit vaccines.

## Materials and Methods

### Mice

The Thomas Jefferson University Institutional Animal Care and Use Committee has approved these studies. Mice were housed in microisolator cages with free access to food and water and were maintained in a specific pathogen-free facility. Wild-type (C57BL/6J; stock no. 000664), TLR4^−/−^ (stock no. 029015), MyD88^−/−^ (stock no. 009088), and Toll/IL-1R domain–containing adapter inducing IFN-β (Trif)^−/−^ (stock no. 005037) mice on a C57BL/6 background were purchased from The Jackson Laboratory (Bar Harbor, ME). Age-matched (8- to 10-wk-old) mice of both sexes were used for all experiments.

### Ags and immunization

ViPS (lot 5, PDMI 158299) isolated from *Citrobacter freundii* strain WR7011 was obtained from the U.S. Food and Drug Administration (Silver Spring, MD). ViPS isolated from *S.* Typhi clinical isolate C652464 was obtained from the International Vaccine Institute (Seoul, Republic of Korea). Typbar TCV was obtained from Bharat Biotech India (Hyderabad, India). Typhim Vi was purchased from Sanofi Pasteur (lot V2A451M). Mice were immunized i.m. with 2.5 μg (50 μl vol) of ViPS in the thigh region of the hindlimb. Blood samples were obtained 0, 7, 14, 21, or 28 d following immunization and stored at −20°C. In some experiments, the phenol-extracted ViPS preparations were admixed with 5 μg of lipid A (Kdo2-lipid A purchased from Avanti Polar Lipids, Alabaster, AL) for immunization.

### *Limulus* amebocyte lysate test

ViPS preparations as well as Typhim Vi and Typbar TCV were diluted to various concentrations in endotoxin-free water and subjected to a *Limulus* amebocyte lysate (LAL) test according to the manufacturer’s instructions (GenScript ToxinSensor chromogenic LAL endotoxin assay kit; GenScript, Piscataway, NJ, lot C53502205). Defined endotoxin standards provided by the assay manufacturer were used to calculate the endotoxin content in the ViPS preparations.

### Stimulation of peritoneal exudate cells

A total of 5 × 10^5^ peritoneal exudate cells/well were plated in a tissue culture–treated, nonpyrogenic, polystyrene 24-well plate, and ViPS from indicated sources or control agonists were added at the indicated concentrations. At 24 h after stimulation, the supernatant was collected, and the IL-6 levels were measured by ELISA according to manufacturer’s instructions (BD Quantikine system).

### TLR, NOD1, and NOD2 ligand screening

TLR/NLR ligand screening was performed using a customized pattern recognition receptor ligand screening service offered by InvivoGen (San Diego, CA; https://www.invivogen.com/custom-tlr-screening). This screen employs HEK293 cells stably expressing a single human or mouse TLR2, TLR3, TLR4, TLR5, TLR7, TLR8, TLR9, NOD1, or NOD2. Stimulation of these TLRs/NLRs are quantified by NF-κB activation, which induces a secreted embryonic alkaline phosphatase (SEAP) reporter. Reagents were tested in triplicate compared with control ligands in a 96-well plate (200 μl total volume) containing the appropriate cells (50,000–75,000 cells/well). Twenty microliters of ViPS or ViPS-containing vaccines, namely Typhim Vi and Typbar TCV, or negative and positive control ligand was added. The media containing QUANTI-Blue reagent detects NF-κB–induced SEAP expression. The OD was read at 650 nm after 16–24 h on a Molecular Devices SPECTRAmax 340PC absorbance detector.

### ELISA

ViPS-specific IgM, IgG, IgG1, IgG2b, IgG2c, and IgG3 were measured by coating 96-well microtiter plates (Nunc MaxiSorp; Invitrogen, Carlsbad, CA) with 2 µg/ml Vi PS purified from *S.* Typhi clinical isolate C652464 (21) in Dulbecco’s PBS overnight at room temperature. All plates were washed and blocked with 1% BSA in PBS (pH 7.2) (blocking buffer) for 2 h at room temperature. Blood from ViPS- or Typhim Vi– and Typbar TCV–immunized mice was diluted to 1:25 and 1:200, respectively, for IgM and IgG detection. These dilutions were based on a linear range of detection. ViPS-specific mouse IgM, IgG, IgG1, IgG2b, IgG2c, and IgG3 levels were interpreted as nanogram per microliter “equivalents” using normal mouse serum standards (Bethyl Laboratories, Montgomery, TX), mouse isotype-specific capture Abs, and horseradish peroxidase–conjugated anti-mouse IgM, IgG, IgG1, IgG2b, IgG2c, and IgG3 as described previously (22).

### Phenol extraction of ViPS

Phenol extraction of ViPS (lot 5, PDMI 158299) was conducted as previously described for other bacterial polysaccharides (23). In brief, 500 μl of triethanolamine (TEA 0.2%)/deoxycholate (DOC 0.5%) solution was added to 1 mg of lyophilized ViPS (lot 5, PDMI 158299). After an overnight incubation at room temperature, 500 μl of TEA/DOC solution containing water-saturated phenol was added and vortexed intermittently for 5 min. The solution was centrifuged for 5 min at 10,000 × *g* at 4°C. The upper, aqueous phase was re-extracted with 0.5 ml of TEA/DOC/phenol. The aqueous supernatant was precipitated with 75% ethanol in 30 mM sodium acetate and the precipitate was washed with 1 ml of ice-cold ethanol, air-dried, and dissolved in 0.5 ml of endotoxin-free water.

### Statistical analysis

Data presented throughout depict pooled data from at least two independent experiments. Statistics were performed using the Prism 9 software program (GraphPad Software, La Jolla, CA), and the statistical tests are indicated in the figure legends.

## Results

**FIGURE 1.**
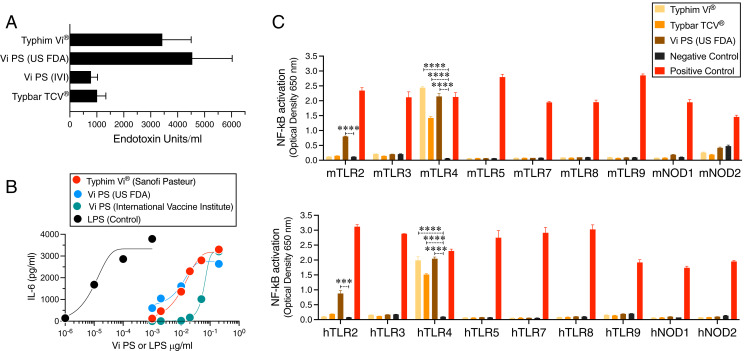
ViPS vaccines stimulate mouse and human TLR4. (**A**) The endotoxin content in each ViPS preparation or vaccines was determined by a chromogenic *Limulus* amebocyte lysate assay. All of the samples were diluted in endotoxin-free water within the range of the known endotoxin standards, and the endotoxin content was determined according to the manufacturer’s instructions. (**B**) Mouse peritoneal exudate cells were incubated for 24 h with various concentrations of ViPS from the three different sources (see *Materials and Methods*) or LPS (Sigma-Aldrich) as a positive control. The levels of IL-6 in the culture supernatant were measured by ELISA. (**C**) HEK293 cells expressing a given TLR or NLR with an NF-κB–inducible secreted embryonic alkaline phosphatase (SEAP) reporter gene were incubated with Typhim Vi (5 μg ViPS/ml lot V2A451M from Sanofi Pasteur), Typbar TCV (5 μg ViPS/ml lot 76B21035A from Bharat Biotech), and ViPS (33 μg ViPS/ml lot 5 PDML158299 from the U.S. Food and Drug Administration). Positive controls were 10^8^ heat-killed *Listeria monocytogenes* cells/ml for mouse/human (m/h)TLR2, poly(I:C) HMW at 1 μg/ml for m/hTLR3, *E. coli* K12 LPS at 100 ng/ml for m/hTLR4, *S. typhimurium* flagellin at 100 ng/ml m/hTLR5, CL307 at 1 μg/ml m/hTLR7, TL8-506 at 1 μg/ml for hTLR8, TL8-506 at 10 μg/ml for mTLR8, CpG ODN 2006 at 10 μg/ml for hTLR9, CpG ODN 1826 at 1 μg/ml for mTLR9, C12-iE-DAP at 10 μg/ml for m/hNOD1, and L18-MDP at 1 μg/ml m/hNOD1. HEK-Blue null cell lines incubated with the above agonists served as negative controls. The medium containing HEK-Blue detection is designed for the detection of NF-κB-induced SEAP expression. After a 16- to 24-h incubation, the OD was read at 650 nm. Statistical analysis used two-way ANOVA with a Sidak multiple comparison test. ***p < 0.001, ****p < 0.0001.

The ViPS used in Typhim Vi and Typbar TCV is extracted from *S.* Typhi strain Ty2, and it therefore may contain trace amounts of LPS (also known as endotoxin), lipoproteins, flagellin, and peptidoglycan fragments during Ag isolation. These microbial-derived molecules activate various members of the TLR family, NOD1, and NOD2. To determine the presence of endotoxin in the ViPS vaccines, ViPS preparations were subjected to an LAL test, which is commonly used and recommended by U.S. Food and Drug Administration to detect endotoxin content in biological samples. Using this test, the presence of endotoxin in all ViPS preparations was readily detectable, and the endotoxin content varied among these samples (Fig. 1A). Furthermore, I found that the unconjugated ViPS preparations from three different sources induced the production of IL-6, a signature cytokine produced by TLR stimulation (24), from mouse peritoneal exudate cells (Fig. 1B). To test a specific involvement of a TLR, as well as NOD1 or NOD2, which recognize bacterial peptidoglycan fragments in the immunostimulatory activity of the ViPS vaccines, a panel of TLR2-, TLR3-, TLR4-, TLR5-, TLR7-, TLR8-, TLR9-, NOD1-, and NOD2-expressing cells with an NF-κB reporter system were screened. In this screen I found that Typhim Vi and Typbar TCV activate mouse and human TLR4 (Fig. 1C).

**FIGURE 2.**
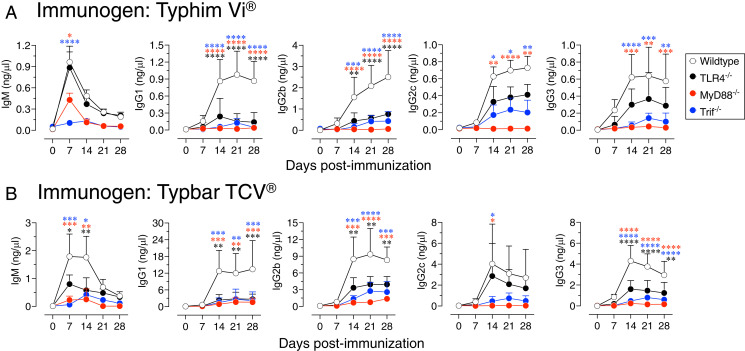
Immunogenicity of Typhim Vi and Typbar TCV is dependent on TLR4, MyD88, and Trif signaling axes. (**A** and **B**) Wild-type (C57BL/6J), TLR4^−/−^, MyD88^−/−^, or Trif^−/−^ (*n* = 6–7) male and female mice 8–10 wk of age were immunized i.m. in the thigh of the hindlimb with 50 μl of (A) Typhim Vi or (B) Typbar TCV vaccines containing 2.5 μg of ViPS. ViPS-specific IgM, IgG1, IgG2b, IgG2c, and IgG3 levels were measure by ELISA. Statistical analysis used two-way ANOVA with a Sidak multiple comparison test. *p < 0.05, **p < 0.01, ***p < 0.001, ****p < 0.0001.

TLR4 signaling is primarily mediated by adaptor proteins MyD88 and Trif. To test the impact of TLR4 activation by the ViPS vaccines in vivo, wild-type, TLR4^−/−^, MyD88^−/−^, or Trif^−/−^ mice were immunized with Typhim Vi and Typbar TCV. Compared to the wild-type, mice deficient in TLR4 showed significantly impaired anti-ViPS IgG1 and IgG2b responses to Typhim Vi and anti-ViPS IgM, IgG1, IgG2b, and IgG3 responses to Typbar TCV (Fig. 2). The most profound impairment of IgM and all IgG isotypes was observed in MyD88^−/−^ mice followed by Trif^−/−^ mice (Fig. 2), indicating that TLR4–MyD88 and TLR4–Trif pathways play nonredundant and partially overlapping roles in the immunogenicity of these ViPS subunit vaccines.

**FIGURE 3.**
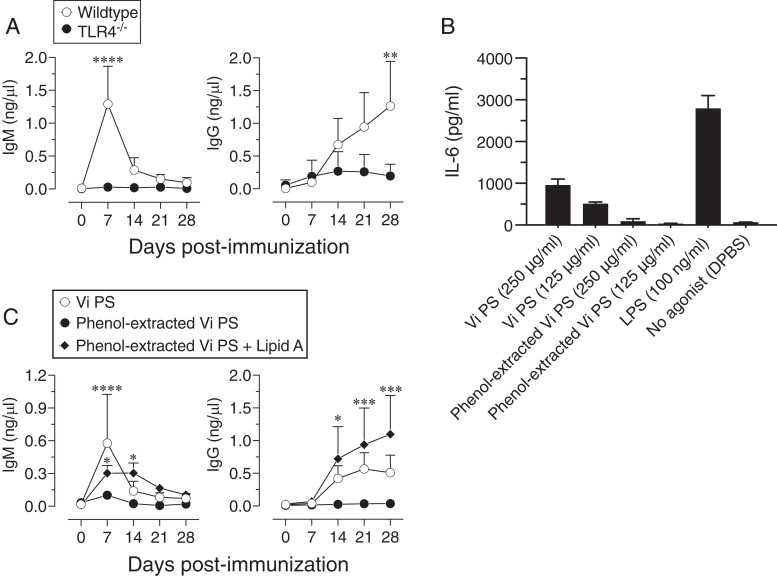
Elimination of immunostimulatory components in the ViPS preparation results in the loss of immunogenicity. (**A**) Wild-type (C57BL/6J) or TLR4^−/−^ mice 8–10 wk of age of both sexes (*n* = 6) were immunized i.m. with 50 μl (2.5 μg) of ViPS (lot 5 PDML158299 from the U.S. Food and Drug Administration), and ViPS-specific IgM and IgG levels were measure by ELISA. (**B**) Peritoneal exudate cells were incubated with indicated stimulants for 24 h, and IL-6 levels in the supernatant were measured by ELISA. (**C**) Wild-type mice were immunized i.m. with 2.5 μg of ViPS and phenol-extracted ViPS in the absence and presence of lipid A (5 μg of Kdo2 lipid A from Avanti Polar Lipids, Alabaster, AL). ViPS-specific IgM and IgG levels were measure by ELISA. Statistical analysis used two-way ANOVA with a Sidak multiple comparison test. *p < 0.05, **p < 0.01, ***p < 0.001, ****p < 0.0001.

Although the ViPS (lot 5, PDMI 158299) stimulated IL-6 and TLR4 as efficiently as Typhim Vi, it also stimulated TLR2 to a lesser extent (Fig. 1A, 1B). However, when this ViPS was used as an immunogen, a significant impairment of anti-ViPS IgM and IgG response was observed in TLR4^−/−^ mice, suggesting that TLR4 activation is the most dominant pathway for its immunogenicity (Fig. 3A). Previously, a phenol extraction procedure of bacterial polysaccharides was shown to eliminate TLR ligands (23). Therefore, ViPS preparation was subjected to this extraction procedure to eliminate the potential presence of TLR ligands. I found that phenol-extracted ViPS not only lost its ability to induce IL-6 production from mouse cells (Fig. 3B), but it also lost its ability to induce an anti-ViPS Ab response (Fig. 3C) to the same extent as the ViPS response seen in TLR4^−/−^ mice (Fig. 3A). Furthermore, the addition of lipid A, a TLR4 agonist to phenol-extracted ViPS, restored anti-ViPS IgM and IgG responses (Fig. 3C). These data indicate that the TLR4 ligand present in the typhoid vaccines is the primary driver for the Ab response to ViPS.

## Discussion

Although the present work demonstrates that an important aspect of associated or carried over TLR4 ligands in the ViPS preparation accounts for the immunogenicity of the ViPS subunit vaccines, it is important to note that this level of endotoxin is within the acceptable limits allowed by regulatory agencies. The level of endotoxin can vary from lot to lot within a given vaccine (20), and vaccine manufacturers often acknowledge the level of endotoxin in their vaccines. All of the approved vaccines, despite having various levels of endotoxin, are safe across all ages. In fact, the safety, immunogenicity, and efficacy of Typbar TCV was consistently shown in three different clinical trials in Nepal, Bangladesh, and Malawi (10, 12–14).

Although I have demonstrated TLR4 ligand activity in Typhim Vi or Typbar TCV, it is possible that the other TLR ligands may also be present in these vaccines. The amount of ViPS itself is 50 μg/ml in these vaccines, and the levels of other TLR ligands could be too low to be detectable in the in vitro assays (e.g., see Fig 1C). The ViPS sample obtained from the U.S. Food and Drug Administration is a lyophilized preparation where the ViPS can be adjusted to a desired concentration. When testing this preparation at an ∼6-fold higher concentration (330 μg/ml), the presence of TLR2 ligand activity was detectable (Fig. 1C). This suggest that Typhim Vi or Typbar TCV may also contain other TLR ligands. In support of this, I found that the induction of certain Ig isotypes was much more affected in mice deficient in MyD88, an adaptor required for many TLRs, including TLR2, than those mice deficient only in TLR4 (Fig. 2).

Immunogenic protein contaminants in pneumococcal polysaccharide subunit vaccines have been observed in humans immunized with Pneumovax 23 and Prevnar (25). In mice the Ab response to pneumococcal polysaccharides is dependent on the presence of ligands that stimulate TLR2 (26). Because *Streptococcus pneumoniae* is a Gram-positive bacterium, the associated TLR ligand is likely to be lipoproteins, rather than LPS. Unlike the TCVs, the pneumococcal conjugate vaccine (but not the unconjugated vaccine Pneumovax 23) contains alum as an adjuvant. Recently, it was shown that incorporation of a mixture of adjuvants that activate TLR, a C-type lectin receptor, and squalene enhanced Ab responses to Pneumovax 23, suggesting the utility of adjuvants in unconjugated pneumococcal polysaccharide vaccines may improve the immunogenicity of unconjugated vaccines (27). In fact, addition of an adjuvant mixture significantly augmented the protective efficacy of Pneumovax 23 against lethal respiratory pneumococcal challenge (27).

Despite the presence of TLR4 ligand in Typhim Vi, the anti-ViPS response is short-lived in human adults (7). In contrast, a single-dose Typbar TCV vaccine gives a significantly heightened and durable Ab response with ∼80% efficacy, demonstrating the superiority of the immunogenicity of conjugate vaccines (10, 12–14). Vaccine manufacturers often indicate the levels of endotoxin, which is below the limits recommended by regulatory authorities. Because the level of “carried over” endotoxin in bacterial polysaccharide vaccines cannot be controlled from lot to lot, as seen for other bacterial polysaccharide subunit vaccines (20), incorporating a defined amount of TLR4 ligand–based adjuvant in typhoid and other bacterial subunit vaccines might make all vaccines equally immunogenic regardless of their intrinsic antigenicity, the carrier protein used for conjugation, or the Ag isolation process, and maximize the polysaccharide subunit vaccine immunogenicity and efficacy.
